# Disinformation, Psychosocial Vulnerability, and Media Trust in the Digital Era: Implications for Health Behaviour and Societal Resilience

**DOI:** 10.3390/healthcare14081089

**Published:** 2026-04-20

**Authors:** João Miguel Alves Ferreira, Vaitsa Giannouli, Sergii Tukaiev

**Affiliations:** 1 Institute of Pharmacology and Experimental Therapeutics, Faculty of Medicine, University of Coimbra, 3004-531 Coimbra, Portugal; 2 Coimbra Institute for Clinical and Biomedical Research (iCBR), Faculty of Medicine, University of Coimbra, 3004-531 Coimbra, Portugal; 3 Center for Innovative Biomedicine and Bio-Technology (CIBB), University of Coimbra, 3004-531 Coimbra, Portugal; 4 Department of Psychology, Democritus University of Thrace, 68300 Didymoteicho, Greece; 5 Faculty of Communication, Culture, and Society, Institute of Public Health, Università Della Svizzera Italiana, 6900 Lugano, Switzerland; 6 Higher Institute of Science Education and Technology, Taras Shevchenko National University of Kyiv, 01033 Kyiv, Ukraine

**Keywords:** disinformation, media trust, psychosocial vulnerability, health behaviour, digital platforms, algorithmic amplification, risk perception, social resilience, political polarisation, cognitive–emotional processing

## Abstract

Disinformation, amplified by digital platforms and algorithmic distribution systems, represents a growing challenge for media trust, public health communication, and societal stability. This narrative literature review examines disinformation through an integrative psychosocial perspective, focusing on how patterns of exposure interact with individual vulnerability factors—including education, political beliefs, social identity, personality traits, and emotional responses to uncertainty—to influence the processing and acceptance of misleading information. The review synthesises interdisciplinary evidence on how algorithmic amplification and emotionally salient content increase susceptibility to disinformation and shape risk perception, health-related decision-making, and preventive behaviours. Findings indicate that repeated exposure to false or misleading information reinforces perceived credibility through familiarity effects, contributes to declining trust in institutional sources, and intensifies social and political polarisation. Disinformation is therefore conceptualised not only as an informational problem but also as a psychosocial process affecting emotional regulation, cognitive evaluation, and collective responses to crises, particularly in public health contexts. The analysis further highlights a recursive feedback loop in which reduced media trust increases vulnerability to subsequent disinformation, with broader implications for democratic participation and social cohesion. Mitigation strategies discussed include media literacy initiatives, critical thinking education, platform governance, regulatory approaches, and interventions targeting psychosocial drivers of susceptibility.

## 1. Introduction

In the digital era, disinformation (intentional deception) has become an omnipresent phenomenon, undermining trust in the media and the integrity of contemporary democracies. Commonly conceptualised as the deliberate dissemination of false or misleading information within digitally mediated environments, disinformation is in contrast to misinformation (unintentional deception) and malinformation (the use of true, accurate, or factual information to inflict harm). Disinformation has emerged as a structurally embedded feature of contemporary communication ecosystems, and it has a profound impact on public perception, eroding the credibility of journalists and media institutions [1]. Conceptually, this review adopts an integrative psychosocial framework in which digital environments and algorithmic amplification shape patterns of exposure to disinformation, psychosocial vulnerability factors (e.g., education, social identity, political beliefs, and personality traits) influence cognitive and emotional processing, and these processes jointly affect media trust (that is a complex construct composed of multiple dimensions such as trust in legacy news, social media, government health agencies, scientists and clinicians), risk perception, and broader health-related behaviours. Within this dynamic cycle, declining trust further increases susceptibility to subsequent disinformation, reinforcing a recursive feedback loop with broader societal and democratic implications. This article explores how disinformation, facilitated by its rapid spread through digital platforms and social networks, affects trust in the media and examines the psychosocial factors that render individuals vulnerable to these false narratives. Digital transformation, associated with infrastructure, device penetration and information digitisation, has fostered global phenomena of concern such as “fake news” [2]. Social media algorithms, designed to maximise engagement, often promote sensationalist and polarising content, exacerbating the spread of disinformation. This environment creates filter bubbles and echo chambers, where individuals are exposed predominantly to information that reinforces their pre-existing beliefs, increasing polarisation and widespread distrust. In addition, factors such as educational level, political beliefs and social identity play a crucial role in susceptibility to disinformation [3]. However, existing research often examines disinformation either from a communication perspective or a public health perspective in isolation, leaving a gap in integrative analyses that consider psychosocial vulnerability, health perception, and media trust as interconnected processes.

To address this complex cross-cultural problem, it is essential to implement media literacy strategies, strengthen fact-checking initiatives/critical thinking and promote transparency in the algorithms of digital platforms not only in healthy individuals, but also in healthcare settings and more specifically in healthcare professionals [4] as well as in patients with different types and severity of diseases across the lifespan [5]. This article analyses these approaches and other possible interventions to mitigate the effects of disinformation, restore trust in the media and safeguard the informational integrity and psychosocial resilience of contemporary societies.

## 2. Study Methodology

This study adopts a narrative literature review approach to examine the relationship between disinformation and health, with particular attention to psychosocial impacts and mitigation strategies. Although this paper is a viewpoint, it is based on the narrative review approach, and this was chosen to allow a broad, interpretative and interdisciplinary exploration of existing scholarship, enabling the integration of findings from communication studies, social psychology, and public health. This approach is particularly appropriate in emerging interdisciplinary fields where conceptual integration and theoretical synthesis are necessary to understand complex psychosocial and behavioural dynamics that cannot be captured through strictly systematic aggregation.

The relevant literature was identified through searches conducted in PsycINFO, PubMed, EMBASE, Web of Science, Cochrane, Google Scholar, and CINAHL. The search combined terms such as “disinformation,” “fake news,” “health perception,” “public health,” “media literacy,” “social identity,” “algorithmic amplification,” and “fact-checking.” The time frame of the literature search was January 2025–January 2026, 5920 articles were retrieved in total, while those articles mentioned in this paper were only chosen as relevant. Inclusion criteria were: any type of paper (empirical research, systematic/narrative review articles) published in English from inception to January 2026 regarding any type of health context and population, while the exclusion criteria were published articles that were not included in journals following the peer-review process and articles that were opinion papers, commentaries and letters to the editor and for which the two screeners did not agree on their relevance. Priority was given to peer-reviewed articles published within the past two decades, particularly those addressing disinformation in digital environments and its influence on health perception and preventive behaviours.

Study selection was guided by relevance to the review’s central research questions rather than by a predefined systematic protocol. Emphasis in this conceptual mapping gwas placed on empirical and theoretical works examining psychosocial vulnerability factors, mechanisms of disinformation dissemination, and consequences for health-related attitudes and behaviours. Articles lacking conceptual or empirical grounding were not considered. All articles were screened for title and abstract by two of the researchers and if judged as eligible they were examined on a full-text basis.

The synthesis of the literature followed an interpretative and comparative approach. The selected studies were examined in relation to their main arguments, methodological orientations, and contributions to understanding disinformation and health. Through this process, recurring themes, conceptual convergences, and areas of debate within the literature were identified.

Given the narrative nature of the review, the findings reflect an integrative and critical interpretation of the existing body of research rather than a quantitative aggregation of results. While this approach does not aim at exhaustive coverage or meta-analytic precision, it enables a comprehensive and theoretically informed understanding of the complex interplay between disinformation, psychosocial dynamics, and public health.

## 3. Disinformation and Trust in the Media

Disinformation, defined as deliberately false or misleading information, has become increasingly frequent, particularly in the digital era. This phenomenon significantly affects public perception and trust in the media, generating a cycle of scepticism and distrust with profound consequences, including increased social and political polarisation, erosion of democratic participation and institutional legitimacy [6], distorted risk perception and reduced adherence to public health behaviours [7,8], and the amplification of fear, anxiety and social destabilisation through the rapid spread of emotionally charged false information [9,10].

### 3.1. The Impact of Disinformation on Trust

Studies show that exposure to disinformation can erode trust in media institutions and journalists. Repeated exposure to false information leads people to question the accuracy of all news, regardless of source. This generalized scepticism undermines the credibility of traditional media, making it more difficult for journalists and media institutions to maintain public trust. International bodies have responded with measures, reports, statements and projects to counter its harms, though the responsibility for institutional education remains an unresolved challenge [2].

For example, research by [11] demonstrates that the spread of fake news during the 2016 US presidential elections significantly affected public perceptions regarding media reliability. The authors argue that the dissemination of disinformation via social networks not only influenced voter beliefs but also contributed to general distrust of traditional media.

### 3.2. Mechanisms of Disinformation Dissemination

The rapid spread of disinformation is facilitated by digital platforms and social networks. These platforms use algorithms that prioritise content with high engagement, regardless of accuracy. As a result, fake news—often more sensationalist and emotionally charged—tends to spread faster than fact-based news [10].

The spread of information (e.g., COVID-19 content) on social networks (Twitter, Instagram, YouTube, Reddit, and Gab) from both reliable and questionable sources occurs in similar ways, but disinformation can propagate more rapidly and widely than evidence-based news [12].

This situation is exacerbated by filter bubbles and echo chambers, where individuals are mainly exposed to information reinforcing their pre-existing beliefs. This not only increases vulnerability to disinformation but also further polarises public opinion, fostering an environment of distrust and division. User polarisation shapes news consumption on social networks (e.g., Facebook) and opinion formation, while biases limit choices to a set of sources and accounts [13].

**Figure 1:**
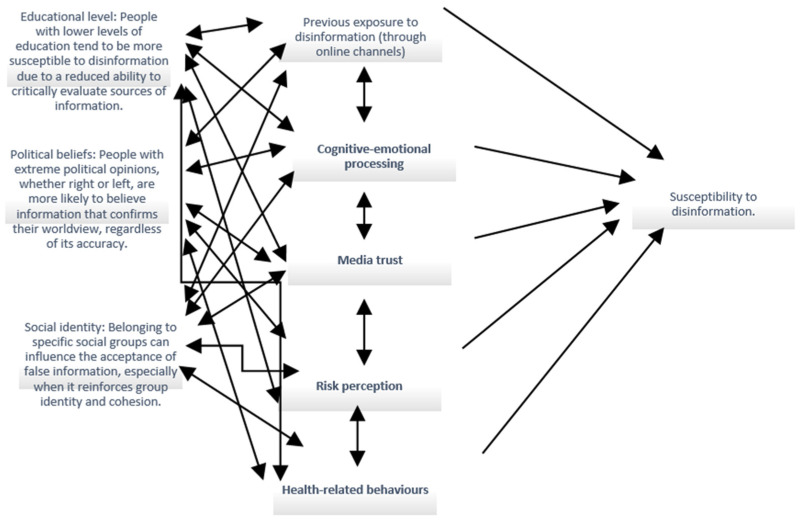
Integrative psychosocial model of vulnerability to disinformation, illustrating how individual characteristics (e.g., education, social identity, political beliefs, and personality traits) interact with exposure patterns and cognitive–emotional processes to influence media trust, risk perception, and health-related behaviours.

Within the integrative psychosocial framework adopted in this review, vulnerability to disinformation emerges from the interaction between exposure dynamics, individual psychosocial characteristics, and cognitive–emotional processing mechanisms, as illustrated in Figure 1.

### 3.3. Psychosocial, Behavioural, and Societal Outcomes of Disinformation

Within the integrative psychosocial framework adopted, the outcomes of disinformation extend beyond misinformation exposure to encompass interconnected psychosocial, behavioural, and societal consequences. The added value of the framework we present here is that in contrast to traditional and existing approaches, this proposed framework uses a wider perspective and aims to use and include all relevant variables/parameters and their interconnections at all levels (cognitive, emotional, social as well as intrapersonal and interpersonal) in the contemporary virtual environment. At the individual level, disinformation can distort risk perception, influence health-related decision-making, and increase anxiety, fear, and uncertainty [9]. At the interpersonal and group level, repeated exposure to polarising and identity-congruent narratives may reinforce echo chambers and intensify social and political polarisation [6]. At the societal level, these processes may undermine institutional trust, weaken democratic participation, and in extreme contexts contribute to social destabilisation and collective conflict. Together, these multilevel effects illustrate how disinformation operates not only as an informational problem but as a psychosocial process with cascading consequences across individual, social, and institutional domains.

**Figure 2:**
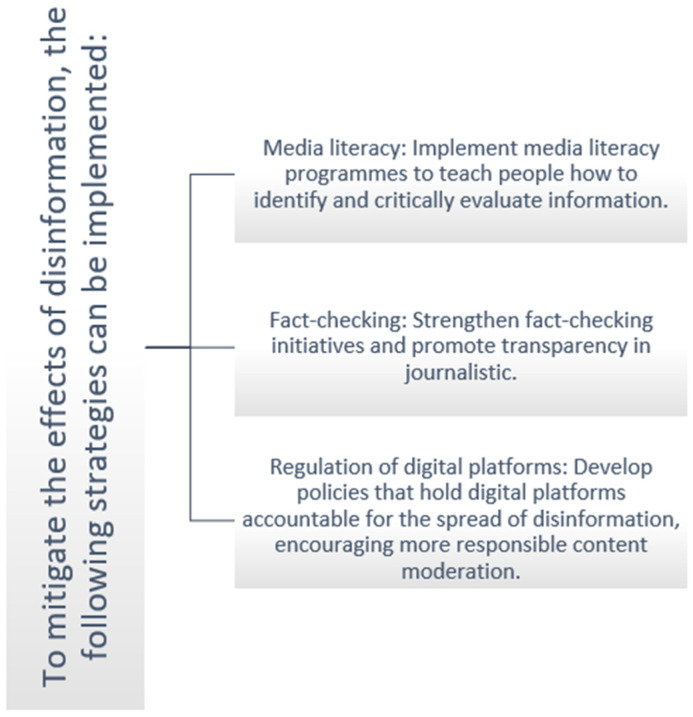
Strategies to Combat Disinformation.

To mitigate the effects of disinformation and restore media trust (Figure 2), various strategies can be adopted.

Disinformation represents a significant threat to trust in media and, by extension, to democratic health. Understanding the mechanisms of disinformation dissemination and the psychosocial factors that increase individual vulnerability is crucial for developing effective strategies to address this phenomenon. By strengthening media literacy, promoting fact-checking, and regulating digital platforms, it is possible to mitigate the effects of disinformation and restore public trust in the media.

## 4. Psychosocial Factors in Vulnerability to Disinformation

Psychosocial factors play a crucial role in individuals’ vulnerability to disinformation. These factors include education, political beliefs, social identity, and previous exposure to disinformation. Understanding these elements is essential for developing effective mitigation strategies and strengthening trust in the media.

### 4.1. Education

Education is a key factor influencing the ability to critically assess information. Studies have found that people with lower educational attainment tend to be more susceptible to disinformation. Education fosters critical and analytical thinking skills that are vital for distinguishing real from false information.

For instance, research by [11] reveals that individuals with higher educational levels are more likely to question information accuracy and check sources before believing them. Education not only improves judgment, but also encourages the appreciation of trustworthy sources.

### 4.2. Political Beliefs

Individuals’ political beliefs are another significant factor influencing vulnerability to disinformation. Those with extreme political views, whether right or left, are more likely to accept information affirming their worldview, regardless of its accuracy—a phenomenon known as confirmation bias.

Research indicates that political polarisation exacerbates disinformation’s spread, as polarised individuals are more likely to accept information reinforcing their ideologies. This is particularly visible in highly polarised settings such as election campaigns or divisive debates [6].

News consumption and opinion formation on social media (e.g., Facebook) are driven by user polarisation; biases reduce selection to a limited set of sources and accounts [13].

Political beliefs have also been shown to determine risk behaviour. Erfei Zhao’s study evaluated preventive and risk behaviour related to COVID-19 infection in the pandemic’s early months based on trust in different media sources. Three groups were identified based on whether participants trusted CNN over Fox News, vice versa, or had similar/no preferences. Conservative individuals trusting Fox News demonstrated less preventive and more risk behaviour for COVID-19 [7].

In China, compliance with conduct rules was associated with trust in central state media and WeChat, whereas reliance on local media and the Weibo network negatively impacted compliance with restrictions [8]. Compliance with public health measures correlated with trust in government, as shown by an online survey of Slovenian adults [14].

### 4.3. Social Identity

Social identity refers to an individual’s sense of belonging to a specific group, which can significantly influence the acceptance of false information. People tend to trust information aligning with their group’s beliefs and norms. Accordingly, disinformation reinforcing group identity and cohesion is more readily accepted [15].

Social identity theory posits that people derive identity and self-esteem from their group affiliations. When false information strengthens this association, individuals are more likely to accept it uncritically. This is particularly problematic in closed communities or groups with strong social ties, where disinformation can spread swiftly and be widely adopted [16].

### 4.4. Personality

Among the reasons for the growth of false information on social media are users’ personality traits, preferences, and psychological characteristics [13]. People are susceptible to conspiratorial thinking and self-deception [17]. The ability to detect the veracity of information, as well as disinformation, is related to emotional intelligence and educational level [18]. Media literacy and critical thinking are fundamental to reducing trust in COVID-19 disinformation [19].

Perception and response to information during epidemics are largely determined by personality factors such as intolerance of uncertainty [20]. Higher anxiety from uncertainty in pandemics increases the likelihood of misinterpreting information and reduces critical evaluation capacity [21]. Intolerance of uncertainty contributes to stress and burnout [22], and burnout negatively affects perception of information emotionality [23].

During the COVID-19 pandemic, conspiracy theories flourished in contexts marked by genetically determined anxiety, powerlessness, perceptions of socio-economic consequences, complex causes and inadequate solutions, as well as modern media [24]. A study by Jakub Šrol in Slovakia found that support for conspiracy theories in the early pandemic was linked to distrust of government responses, increased anxiety and lack of control, amplifying the perceived danger and risk of COVID-19 [25]. Conspiracy theories hinder joint action, distort open debate, and sow doubt [24]. High intolerance of uncertainty in people who do not believe in conspiracies aids in risk assessment concerning SARS-CoV-2 [20].

False news, disinformation and rumours spread via digital media become sources of panic, anxiety and fear, increasing mental illness risk [9]. Beliefs shaped by disinformation influence coping strategies during pandemics [21]. Deceptive information about COVID-19 increases societal uncertainty [21]. Beyond cognitive and personality traits, emerging theoretical perspectives suggest that subjective experiences of existential disconnection or chronic boredom may increase openness to simplified or emotionally charged narratives, as individuals seek meaning, coherence, or psychological stimulation in uncertain informational environments [26,27,28].

Deliberative decision-making is influenced by emotions. The patterns of decision-making can be explained by the competitive neuro-behavioural decision systems (CNDS) theory. According to CNDS, the impulsive system is related to the saliency and valuation of immediate rewards (immediacy bias) and leads to suboptimal decisions favouring short-term benefits. Imbalanced regulation between executive and impulsive neural systems negatively affects health behaviours [29]. Vaccine acceptance as a socially significant action can be viewed as classic decision-making, involving five basic processes: (1) learning (updating representation, valuation, and action selection), (2) representation (identifying viable actions), (3) evaluation (appraising each action), (4) action selection, and (5) outcome evaluation [30].

### 4.5. Previous Exposure to Disinformation

Repeated exposure to disinformation increases vulnerability. When people are exposed again and again to false information, they tend to accept it as true—a phenomenon known as the “illusory truth effect”. Familiarity with information heightens perceived veracity, regardless of accuracy [31].

Ref. [32] found that repeated exposure to false statements increases belief in them, even among individuals with knowledge contradicting those claims. Repeated disinformation creates a persistent impression that is difficult to correct, even when accurate information is later provided.

### 4.6. The Whole Is Greater than the Sum of the Parts

Vulnerability to disinformation is shaped by a complex interplay of psychosocial factors, such as education, political beliefs, social identity, and repeated exposure to false information. Understanding these factors is crucial for developing effective interventions to reduce the spread of false information and restore media trust. Strategies such as media literacy programmes, promoting critical thinking, and fact-checking initiatives are key to mitigating disinformation’s negative impacts [33].

## 5. Digital Media and Social Networks

Digital platforms and social networks have transformed how we consume and disseminate information. While they have democratised access to information, they have also enabled the rapid and widespread spread of disinformation, making the problem even more complex and challenging.

In Brazil, the main sources of fake news originate from social networks: WhatsApp, Facebook and Instagram [34]. A study in Spain found that social networks and instant messaging systems are the main transmitters of fake news. This “primacy” is due to declining trust in the media, which are no longer considered reliable sources [35]. Motivating social network users to share, communicate, and seek information contributes to the spread of COVID-19 disinformation [36]. In Italy, during the pandemic, an interesting phenomenon was that trust in social networks was lower than in official health websites [37].

### 5.1. Again, the Impact of Disinformation on Trust

Social media algorithms play a crucial role in the spread of disinformation. They are designed to maximise user engagement by promoting content that generates more interactions, such as likes, shares and comments. Often, this content is sensationalist and polarising, as strong emotions like anger and fear generate greater engagement than neutral or positive information [10].

This algorithmic promotion creates a favourable environment for disinformation. Fake news, typically more emotional and surprising than the truth, spreads quickly, reaching many people in a short time [10]. This can generate a vicious cycle where disinformation is continually amplified, while corrections and accurate information receive less visibility.

### 5.2. Filter Bubbles and Echo Chambers

In addition to promoting polarising content, social media algorithms also create filter bubbles and echo chambers. Filter bubbles arise when algorithms personalise content based on users’ past behaviours and preferences, limiting exposure to diverse information. Users are therefore likely to see only content supporting their existing beliefs, isolating them from opposing viewpoints [38].

Echo chambers reinforce this dynamic, as users primarily interact with people who share their views. This further entrenches pre-existing beliefs and increases polarisation, making individuals more susceptible to disinformation that confirms their worldview. This polarised environment makes it harder to correct false information, as people are less likely to accept contradicted beliefs.

### 5.3. Viral Spread of Disinformation

The viral nature of social networks means that disinformation can spread exponentially. A single false post can be shared thousands of times in hours, reaching a vast and diverse audience. The speed at which disinformation spreads makes it difficult for fact-checkers and media organisations to correct false information effectively and promptly [39].

Studies show that fake news spreads significantly faster and reaches more people than truthful news. For instance, Ref. [10] found that false information on Twitter spreads six times faster than the truth. This swift spread not only misleads individuals but also contributes to widespread distrust in the media and the information they provide.

### 5.4. Impact on Trust in the Media

The proliferation of disinformation on social networks directly affects people’s trust in the media. Repeated exposure to false information leads individuals to question the accuracy of all information, including that from reliable sources, fostering general scepticism.

Disinformation can also damage the reputation of traditional media when fake news is mistakenly attributed to credible sources. This is particularly problematic as boundaries between different types of media become increasingly blurred. The confusion between legitimate journalism and disinformation can further weaken trust in media institutions.

**Figure 3:**
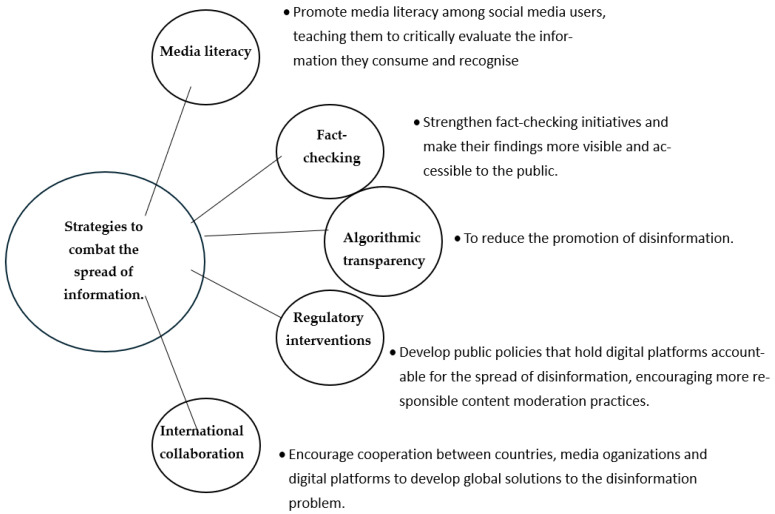
Integrated Strategy to Mitigate the Spread of Disinformation.

To address disinformation on digital platforms (Figure 3), several strategies can be implemented.

Digital platforms and social networks play a central role in spreading disinformation, exacerbated by algorithms that promote polarising content and filter bubbles that isolate individuals within echo chambers. This accelerating dynamic presents a major challenge for media trust. Educational, regulatory and collaborative strategies are essential to mitigate disinformation and restore public trust in the media.

## 6. Conclusions

In conclusion, disinformation presents a growing challenge to trust in the media and democratic stability. The interplay of digital platforms and social networks amplifies the spread of false information, exacerbated by algorithms that promote polarising content and filter bubbles that insulate individuals. This dynamic not only accelerates disinformation’s spread but also undermines public trust in traditional media institutions, creating a damaging cycle of scepticism and disinformation. Psychosocial factors such as education, political beliefs, and social identity significantly influence individual vulnerability to disinformation [40]. From a broader psychosocial perspective, strengthening resilience against disinformation may also require addressing underlying experiences of existential uncertainty, meaning disruption, and subjective disengagement that increase vulnerability to simplified explanatory narratives [26,27,28]. The lack of awareness regarding digital identity, social media consumption, and fake news underscores the urgent need for educational intervention [2]. Media literacy is crucial, equipping people with critical thinking skills needed to distinguish between true and false information. Robust fact-checking and greater algorithmic transparency are also essential for reducing the promotion of disinformation on social networks. Effectively addressing disinformation requires coordinated efforts, including regulatory policies that hold digital platforms accountable and foster international collaboration. Only through an integrated approach combining education, regulation and technological innovation can trust in the media be restored and the integrity of the public sphere and democratic processes safeguarded.

## 7. Scientific Implications and a Critical Reading of the Findings

The scientific implications of studying disinformation and its interaction with the media, digital platforms and psychosocial factors are vast and multifaceted, encompassing research fields such as communication, psychology, data science and public policy. The spread of disinformation highlights the urgent need to revise and update communication and media theories, as traditional models of information flow and credibility face significant challenges in the digital era. Future research should explore new ways of measuring and understanding the effectiveness of disinformation mitigation strategies and the resilience of audiences in the face of false information.

Furthermore, the analysis of social media algorithms and their impact on disinformation can provide valuable insights into how modern communication networks influence public perception. Studying the psychosocial factors affecting vulnerability to disinformation, such as educational attainment, political beliefs and social identity, has important implications for social and behavioural psychology. Understanding why and how certain groups are more susceptible to disinformation can inform the development of psychological and educational interventions.

Research on the “illusory truth effect” and confirmation bias can also help create strategies to enhance critical thinking and cognitive resilience, offering ways to reduce polarisation and misinformation’s impact. Innovations in fact-checking and algorithmic transparency rely on ongoing technological advancement and more sophisticated methods. Data science plays a critical role in combating disinformation, with tools and algorithms for detecting fake news, analysing dissemination patterns and evaluating intervention effectiveness benefiting from interdisciplinary research. Studies on the effectiveness of fact-checking initiatives and the ability of algorithms to prioritise reliable information are vital to developing technological solutions that curb disinformation’s spread.

Regulating digital platforms and holding disinformation distributors accountable requires rigorous scientific analysis of legal and ethical implications. Research can help shape effective and fair regulations protecting information integrity without compromising freedom of expression. Studies on best regulatory practices and content moderation are essential for developing balanced policies for digital safety and freedom.

Promoting media literacy and critical thinking is fundamental for reducing disinformation susceptibility. Research can guide the development of educational programmes and curricula that teach essential skills for critically assessing information. Evaluating the effectiveness of such educational approaches and developing new teaching strategies can enhance media literacy and better prepare individuals to confront disinformation’s challenges.

These scientific implications underscore the need for a multidisciplinary approach to understand and address the complex challenges posed by disinformation. Advancing research in communication, psychology, technology and public policy is essential to mitigate disinformation’s impact and strengthen trust in media institutions and democratic processes.

A critical reading of the findings shows that due to methodological and cultural differences, a direct comparison across studies is difficult, even impossible. Although the vast majority of the published material comes from the western cultural context, it is not possible to discuss contradictory findings given the differences in the applied methodologies, the conceptualizations of the variables and the ways in which these variables were actually measured. Despite that, the unique contribution and novelty of the proposed framework is that readers can gain new insights regarding the importance of long-neglected variables in the proposed conceptual model, and at the same time gain a new perspective on how practice should be transformed given the importance of the proposed variables and especially their interconnections following a holistic approach, something that could shape the future research agenda on this emerging topic. Although there is a plethora of implications, we believe that clinical settings, patient behavior, healthcare professionals, risk communication, vaccine uptake, and adherence are all influenced by a multivariable approach that clearly recognizes the complexity of the phenomenon.

## 8. Limitations and Future Research

This study is based on a narrative literature review and therefore reflects an interpretative synthesis of selected scholarly contributions rather than a systematic or exhaustive mapping of all available evidence. Although efforts were made to identify relevant and high-quality sources, the selection of studies was guided by thematic relevance and conceptual contribution, which may introduce an element of subjectivity inherent to narrative approaches.

Given the absence of a formal systematic protocol, the review does not aim to provide quantitative aggregation or meta-analytic precision. Instead, it offers a theoretically informed integration of research across disciplines. Consequently, some relevant studies may not have been included, particularly those published in languages other than English or in less accessible regional journals.

In addition to methodological considerations related to the review design, the field of disinformation research itself presents structural challenges. Many empirical studies rely on data from specific digital platforms or limited geographic contexts, which may restrict generalisability. The lack of transparency in social media algorithms further constrains researchers’ ability to fully understand how information is prioritised and disseminated. Moreover, the absence of a universally accepted definition of disinformation can generate conceptual variability across studies, complicating comparison and synthesis.

The dynamic and evolving nature of digital communication ecosystems also makes it difficult to assess long-term impacts on media trust and health-related behaviours. Rapid technological change may outpace empirical investigation, requiring continuous theoretical and methodological adaptation.

Future research would benefit from comparative cross-cultural studies, greater transparency in platform governance, and longitudinal designs capable of assessing enduring psychosocial and behavioural effects. Further evaluation of media literacy interventions, regulatory frameworks, and algorithmic accountability mechanisms is essential. Interdisciplinary collaboration between communication scholars, psychologists, public health researchers, and data scientists will remain crucial for advancing understanding of disinformation and its societal implications.

## Data Availability

No new data were created or analyzed in this study.
